# The Effect of Electrical-Stimulation-Induced Emotion on Time Perception: A Time-Reproduction Task

**DOI:** 10.3390/ijerph192416984

**Published:** 2022-12-17

**Authors:** Chunni Zhao, Qing Zeng

**Affiliations:** 1 School of Marxism, Foshan University, Foshan 528011, China; 2 School of Marxism, Jinan University, Guangzhou 510632, China

**Keywords:** duration perception, time-reproduction task, electric stimulation, arousal

## Abstract

Duration cognition refers to an individual’s cognition for the duration of a given stimulus. Previous studies have explored the effect of emotions on duration perception; however, the results remain controversial. To explore the characteristics of college students’ time perception under electrical stimulation, this study used a time-reproduction task and a within-subject design with electrical-stimulation conditions and target duration as independent variables. Additionally, this study used the average temporal reproduction and the reproduction coefficient of variation as dependent variables; the subjective arousal degree, value, and electrical activity under electric stimulation were recorded simultaneously. The results indicated a significant main effect of electrical stimulation. Compared to non-electrical stimulation, the average temporal reproduction of participants under electrical stimulation was significantly shorter. Additionally, the interaction between electrical stimulation and target duration was significant. Furthermore, with the increase in the target duration, the shortening degree of the average temporal reproduction under the electrical stimulation increased significantly. Additionally, the participants’ subjective arousal with electrical stimulation was higher than that without an electrical shock, and the valence with electrical stimulation was lower than that without electrical stimulation. These results suggest that the emotions induced by electrical stimulation increase the internal-clock speed, which leads to the relative overestimation of time perception.

## 1. Introduction

Duration cognition mainly refers to an individual’s cognition of the duration of a given stimulus, which may range from milliseconds to hours [1]. Duration perception, also known as subjective present, falls within the scope of duration cognition. It refers to an individual’s perception and estimation of a duration of 2–3 s on average (no longer than 5 s) [2].

In the past 10 years, emerging research evidence has suggested that an individual’s emotions affect their time perception. Previous studies mainly used emotion-eliciting faces [3,4,5,6,7], emotion-eliciting scenes [8,9,10,11], and emotion-eliciting sounds [12,13] to explore how emotions affect duration perception. However, how emotions affect time perception has been controversial. This inconsistency may be due to the emotional effect of time perception being based on different hypotheses of the timing-processing mechanism of emotional stimuli.

According to the Scalar Expectation Theory (SET), the effect of emotions on duration perception is mainly derived from two mechanisms [14]: the attention-switch mechanism and the pacemaker-acceleration mechanism. Under the attention-switch hypothesis, the attention switch is turned off at an early stage, while an emotional effect is produced. An extra constant number of pulses are added to those already processed and accumulated during the target duration. Furthermore, there is an addition effect, in that the number of pulses added remains the same regardless of the duration of stimuli. According to the internal-clock model, perceived duration is based on the number of pulses collected in the pacemaker; thus, the more pulses that are accumulated, the longer the perceived duration is. A “multiplication” relationship exists between emotional effect and duration effect, in that, as the duration of the stimulus increases, the number of extra pulses added increases at an accelerated rate.

Previous studies based on the SET hypothesis revealed the effect of emotions on duration perception. However, two points in these studies are worth discussing. First, the stimuli used were subject to limitations regarding duration. The stimuli usually lasted under 2 s, which resulted in a time window that was too narrow to comprehensively identify the effect of emotions. Droit-Volet [15,16] used a temporal bisection task (0.4–0.8 s, 0.8–1.6 s; 0.2–0.8 s, 0.4–1.6 s) to examine the effect of emotions on duration perception. Their results indicated that emotions had a multiplying effect on participants’ estimations of duration, which supports the clock speed hypothesis. However, due to the durations selected in these studies being within 2 s and, therefore, not long enough to cover the entire range of duration perception, it was impossible to comprehensively examine the mechanism underlying the effect of emotions on duration perception. In contrast, electrical stimulation was also used in two earlier studies to examine longer durations (>5 s) [17]. These studies found that the participants estimated the duration of the stimulus to be longer with electrical stimulation than without electrical stimulation. However, Falk and Bindra only tested one duration (15 s) [18], and Hare [17] tested two durations (5 s and 20 s). As the direct consequence of the studied duration (>5 s) was beyond the range of time-distance perception, these studies failed to reveal the mechanism underlying the effect of emotions on duration perception.

Second, the stimuli (such as images) used only elicited transient emotions (i.e., the participant’s responses to the stimuli that were rapidly decreased). For example, studies in which durations exceeding 2 s were used found that, in the case of long durations, the prolongation effect of emotions continued to be weakened rather than enhanced [8,19]. This appears to be inconsistent with the internal-clock-acceleration hypothesis. However, the weakened effect of emotions on the duration perception may point to a mechanism based on dynamic changes in arousal [3]. Simultaneously, the physical characteristics of emotion-eliciting stimuli (color in pictures or rhythm in sounds) may interfere with time processing and, consequently, change the effect of emotions on duration perception [20]. In this regard, procedures that can continuously induce emotions should be adopted, while interference from the physical features of the emotional stimuli should be excluded. Therefore, due to the transiency and variability of the effect of emotion-eliciting stimuli, the best way to clarify the multiplication (clock speed) and additional (the turning off of the attention switch) effects is to examine the effect of fully induced emotions on an individual’s perception of multiple long durations (>2 s). Electrical stimulation was applied to induce sustained emotional experience [21] and continuously induce emotional experience [22,23]. This solved the phenomenon of the emotional effect significantly being reduced under long-term utility (>2 s); however, it had no physical characteristics, which were used in previous time-perception studies [21].

This study aimed to examine the effect of emotions induced by electrical stimulation on the duration perception in a wider range (2–5 s). A time-reproduction task, the Self-Assessment Manikin questionnaire, and electrodermal activity (EDA) were used to address these research questions.

The innovation of this study resides in the following aspects: (1) This study used electrical stimulation to induce emotion, which eliminated the impact of the physical characteristics of emotional stimulus and changed the lack of a time limit for emotional effectiveness. (2) In this study, five replicating time intervals were selected to examine the effect of emotions on time-interval perception. The shortest time interval (500 ms) among the five time intervals was the lowest possible value in a wide range (2–5 s), which exceeded the simple reaction time and was near the selective reaction time [24]. The longest time interval (4500 ms) in the five time intervals was the highest possible value in a wide range (2–5 s), which was near the upper-end of time-interval perception but did not exceed 5 s [25]. (3) The My Assessment of Human Models Scale [26] was used to assess the level of subjective arousal and emotional potency, and the electrical activity index [27] was used to assess the physiological indicators of arousal.

This study predicted that reproduced durations would be shorter with electrical stimulation than without electrical shocks and that their lengths would depend on the target durations.

## 2. Materials and Methods

### 2.1. Participants

G-power 3.1 (alpha level 0.05, power 0.8, effect size 0.3) [28] was applied to calculate the total number of participants required, which indicated that 18 participants were required. To enhance the power and consider actual and other factors in previous studies, 25 participants were recruited (13 females). The average age was 21.28 years old (SD = 0.44). The sample consisted of Chinese university students who were studying psychology as a second major. All participants signed an informed consent form. After completing the experiment, participants received a partial grade in a psychology course and RMB 60 as a participation reward. This study was approved by the Research Ethics Review Board of Foshan University (Approval Number: 2021-1-015).

### 2.2. Stimuli and Measures

#### 2.2.1. Equipment and Materials

The equipment included two computers. E-prime 2.0 was used to generate procedures and record data. The timing stimuli were gray ellipses and squares on the computer screens. The participants pressed “Enter” on the keyboard with their dominant hand for short and long responses.

#### 2.2.2. Electrodermal Activity (EDA)

Electrical stimulation was applied to the participants using a multi-channel electrical stimulator, which entailed placing the Velcro strap of an electrode on the middle finger of their dominant hand. The intensity of the shock was adjusted by controlling the voltage value of the electrical stimulator. A common 1–4 s latency window (i.e., 1–4 s after a stimulus was introduced) was selected, with a standard minimum amplitude of 0.05 s [29]. For analysis purposes, the participants’ EDA responses were corrected based on their EDA values prior to stimulation. These data were subsequently converted to square roots to standardize response amplitude data [30] and were averaged for each type of test. Each participant was required to complete an electrical-stimulation-threshold procedure developed by Crockett [31] before the experiment, which ensured that a gradually enhanced electrical stimulation matrix could be obtained. The procedure obtained 20 different voltage intensities (from weak to strong) generated by each participant based on their perceptions. Before the formal experiment, each participant measured the sensory threshold of shock intensity and subjectively assessed the psychological perception of shock intensity through a nine-point rating scale (one meant no aversion; nine meant very disgusted). The score voltage intensity of the points between one and three was defined as low shock intensity, and the voltage intensity between six and nine points was defined as high shock intensity. Participants selected shock intensity levels ranging from 0.1 mA to 6.4 mA. The additional computer was used to generate electric shocks and record the EDA using LABChart.(ADInstruments Pty Ltd., Bella Vista, Australia)

#### 2.2.3. Subjective Assessments

In this procedure, participants were required to use a nine-point self-assessment manikin questionnaire to assess their emotional valence (from unpleasant to pleasant) and subjective arousal (from calm to very excited) during the test [26].

### 2.3. Experiment Design

We employed a within-subject design to investigate the characteristics of university students’ time perception afterelectrical stimulationor without electrical stimulation, and target duration (500, 1500, 2500, 3500, 4500 ms). Dependent variables included average reproduced duration and coefficient of variation. Average reproduced duration was an average of multiple reproduced durations for each individual under each condition and represented the length of an individual’s subjective reproduced duration. A longer average reproduced duration corresponded to a higher subjective estimate on the individual’s part. The coefficient of variation referred to the standard deviation of multiple reproduced durations for each individual under each condition divided by the average of these reproduced durations. It indicated the variability of the individual’s subjective reproduced durations. The larger the coefficient was, the greater the variability was.

### 2.4. Experimental Procedure

Each participant participated separately in the experiment in a laboratory. During the experiment, the participants sat in front of the monitor and were 70 cm away from the screen. The duration-reproduction task consisted of an exercise phase and a formal experiment phase.

**Figure 1:**
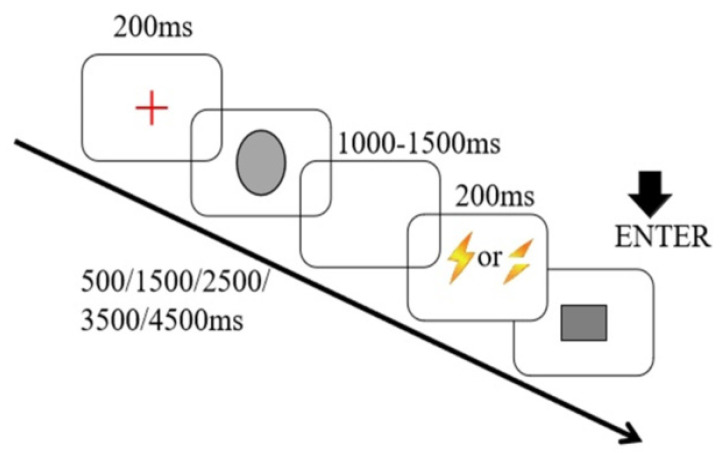
A depiction of the exercise phase and formal experiment in the duration-reproduction task.

During the exercise phase, the stimulation process was as follows: First, a red “+” appeared on the screen indicating that the test has begun. Following this, a gray ellipse appeared on the screen for a period of time (500, 1500, 2500, 3500, 4500 ms). After an interval of 1000–1500 ms, a gray square appeared. When the participant deemed the duration of the gray square to be equal to that of the ellipse, they immediately pressed “Enter” on the keyboard, and their feedback was shown on the screen. When the reproduced duration was longer than 110% of the corresponding target duration, the feedback would be “Too long”; when it was shorter than 90% of the latter, the feedback would be “Too short”; when it was between 90% and 110%, the feedback would be “Appropriate” [32]. The exercise consisted of one block that included 10 tests (5 target durations repeated twice), and the tests within the block were randomly presented. A participant passed the exercise when more than 80% of the feedback was “Appropriate.” The exercise repeated until the participant passed. At the end of the exercise phase, each participant was presented with two types of symbols on the screen: a yellow lightning bolt and a yellow interrupted lightning bolt (see Figure 1). They were told that a lightning bolt indicated that electrical stimulation would be applied during the subsequent duration-reproduction phase, while an interrupted lightning bolt indicated that there was no electrical stimulation in the subsequent replication phase. During the formal experiment phase, the stimulation process was the same as during the exercise phase, with the exception that no feedback was provided and that a symbol appeared during the interval indicating whether electrical stimulation would be applied. The formal experiment consisted of 8 blocks, each of which included 20 tests (5 target durations repeated twice with electrical stimulation and 5 target durations repeated twice without electrical stimulation). Therefore, a total of 160 tests were randomly presented within each block.

After the duration-reproduction task, the participants were required to complete tests to assess their physiological responses (EDA) and subjective emotions during the time they waited for electrical stimulations.

Regarding the EDA assessment, participants were required to complete 10 tests, including 5 with electrical stimulation and 5 without electrical stimulation. These tests were the same as those in the duration-reproduction task, with the exception that the interval between the two tests was 20 s. To assess the participants’ subjective emotions, they completed 10 tests, including 5 with electrical stimulation and 5 without electrical stimulation.

At the end of each test, participants were required to use a nine-point self-assessment model to assess their emotional valence (from unpleasant to pleasant) and subjective arousal (from calm to very excited) during the test [26].

## 3. Results

### 3.1. Assessment and Analysis of Subjective Emotions

As shown in Table 1, the statistical indicators of the mean and standard deviation of the arousal, valence, and EDA were measured with electrical stimulation and without electrical stimulation.

A two-factor analysis of variance (ANOVA) was performed on the independent variables (including five target durations repeated with electrical stimulation and without electrical stimulation), with average arousal rating as the dependent variable. The main effect of electrical stimulation was found to be significant (F(1, 24) = 229.263, *p* < 0.001, η^2^p = 0.905), and the arousal rating with electrical stimulation (M = 5.152 ± 0.079) was found to be significantly higher than that without electrical stimulation (M = 3.832 ± 0.057). The main effect of target durations and the interaction between target durations and electrical stimulation was not significant (*p* > 0.05). Another two-factor ANOVA was performed on the independent variables (including five target durations repeated with electrical stimulation and without electrical stimulation), with average valence rating as the dependent variable. The main effect of electrical stimulation was found to be significant (F(1, 24) = 22.756, *p* < 0.001, η^2^p = 0.487), and the valence rating with electrical stimulation (M = 4.912 ± 0.087) was found to be significantly lower than that without electrical stimulation (M = 5.552 ± 0.113). The main effect of target durations and the interaction between target durations and electrical stimulation was not significant (*p* > 0.05).

### 3.2. Electrodermal Activity (EDA)

A two-factor ANOVA was performed on the independent variables including electrical stimulation and target durations, with EDA amplitude as the dependent variable. The main effect of electrical stimulation was found to be significant (F(1, 24) = 76.176, *p* < 0.001, η^2^p = 0.760), and EDA amplitude with electrical stimulation (M = 1.325 ± 0.025) was found to be significantly higher than that without electrical stimulation (M = 1.027 ± 0.022). Other effects were not significant (*p* > 0.05). The physiological arousal indicator of EDA amplitude indicated that arousal was significantly higher with electrical stimulation than without electrical stimulation.

### 3.3. Average Reproduced Duration

As shown in Table 2, the statistical indicators of the mean and standard deviation of the average reproduced duration and the coefficient of reproduction variation were measured with electrical stimulation and without electrical stimulation.

A two-factor ANOVA was performed on the independent variables (including five target durations repeated with electrical shocks and without electrical shocks), with average reproduced duration as the dependent variable. The main effect of electrical stimulation was found to be significant (F(1, 24) = 151.577, *p* < 0.001, η^2^p = 0.863) and the reproduced duration with electrical stimulation (M = 2437 ± 13.79) was found to be shorter than that without electrical stimulation (M = 2506 ± 12.85). The main effect of target durations was significant (F(4, 96) = 4378.001, *p* < 0.001, η^2^ p= 0.955) for 0.5 s (M = 498 ± 13.62), 1.5 s (M = 1564 ± 18.94), 2.5 s (M = 2506 ± 22.97), 3.5 s (M = 3467 ± 29.17), and 4.5 s (M = 4322 ± 31.82), with *p* < 0.001. The average reproduced duration increased progressively and significantly. A significant interaction was evident between target durations and electrical stimulation (F(4, 96) = 18.512, *p* < 0.001, η^2^p = 0.435). Further analysis revealed that, at 0.5 s, the reproduced duration with electrical stimulation (M = 491 ± 14.43) was shorter than that without electrical stimulation (M = 506 ± 13.23), with *p* = 0.009. At 1.5 s, the reproduced duration with electrical stimulation (M = 1543 ± 20.97) was shorter than that without electrical stimulation (M = 1586 ± 19.50), with *p* = 0.006. At 2.5 s, the reproduced duration with electrical stimulation (M = 2492 ± 25.89) was shorter than that without electrical stimulation (M = 2520 ± 23.66), with *p* = 0.155. At 3.5 s, the reproduced duration with electrical stimulation (M = 3392 ± 31.19) was shorter than that without electrical stimulation (M = 3542 ± 27.81), with *p* < 0.001. At 4.5 s, the reproduced duration with electrical stimulation (M = 4268 ± 34.82) was shorter than that without electrical stimulation (M = 4375 ± 30.27), with *p* < 0.001.

### 3.4. Coefficient of Reproduction Variation

A two-factor ANOVA was performed on the independent variables (including five target durations repeated with electrical stimulation and without electrical stimulation), with the coefficient of reproduction variation as the dependent variable. The main effect of electrical stimulation was found to be significant (F(1, 24) = 11.810, *p* = 0.002, η^2^p = 0.330), and the coefficient of reproduction variation with electrical stimulation (M = 0.186 ± 0.005) was found to be higher than that without electrical stimulation (M = 0.146 ± 0.006). The main effect of target durations and the interaction between target durations and electrical stimulation was not significant (F(4, 96) = 0.847, *p* = 0.499; F(4, 96) = 0.504, *p* = 0.733)).

## 4. Discussion

This study aimed to examine the influence of emotional effects on time-distance perception over a long duration (2–5 s). The results indicated that the average temporal replication time (2–5 s) of participants under electrical stimulation was significantly shorter than that for without electrical stimulation. In addition, participants’ subjective assessments revealed a lower degree of pleasantness and a higher arousal with electrical stimulation than that for without electrical stimulation. Consistent with participants’ feedback, increased EDA with electrical shocks indicated a threatening physiological response that, in turn, indicated an increase in arousal [33].

These findings suggest that prolongation of perceived durations with electric shocks occurs not only in the range of short durations of hundreds of milliseconds (0.5 s) but also in the range of longer durations beyond 2 s (2–5 s). Therefore, time distortion, which is often observed in the range of short durations (under 2 s), applies to a wider range. This is consistent with the prolongation effect which occurs in humans’ time perceptions of events with a long duration, for example, in estimating the duration of a 3 min video clip of the 9/11 terrorist attack (Anderson et al. 2007) or when a novice skydiver estimates the duration of their first jump [34]. This is consistent with an individual’s experience of time distortion in traumas involving aggression or accidents [35]. Therefore, in a threatening environment, time distortion is intense enough to be observed in different durations. 

Additionally, this study found that emotion-related time distortion increases in intensity simultaneously with the duration of the stimulus. The hypothesis in this study, suggesting that applying electrical shocks within the duration of a stimulus can increase arousal and, therefore, automatically accelerate the internal-clock system, was, thus, verified. The shock automatically activates various fear-related physiological responses (increased heart rate, pupil dilation, muscle contraction), which prepare the entire body for survival (i.e., to avoid an imminent danger), while also automatically accelerating the internal clock through threat awareness [36]. This explanation is supported by the role of the dopaminergic system in timing and emotional processes, with increased levels of dopamine in the brain producing temporary time overestimation. Thus, the acceleration of the internal clock can be seen as an automatic response to general physiological activation in a threat situation [37].

However, previous studies have shown inconsistent results for the impact of emotions on time perception. Some studies have found an interaction between emotion and duration (multiplication effect) in short durations of under 1 s [38]; however, this was not present in other time ranges [39]. Other studies have found that the effect of emotions on time perception decreased after 2 s [8]. In contrast, another study found that an emotion-eliciting scene can sufficiently increase the participants’ arousal such that time distortion not only lasts for a long time (>2 s) but also increases in intensity as the duration of the stimulus increases. Therefore, a hypothesis based on the findings of this study is that the inconsistent findings from previous studies or the absence of sustained effects are due to the emotion-eliciting stimuli (images) used, which could not fully activate continuous increases in arousal levels. The emotions induced by emotion-eliciting images are short-lived and, therefore, subject to change. This makes it difficult to test the prediction from the SET theory regarding the mechanism underlying the effects of emotions on time perception. In other words, emotion-eliciting stimuli other than images are required to further examine how emotions affect the perception of durations (including long ones). The procedures used in this study allowed for the testing of the hypothesis that stated that the interaction between emotions and durations is based on arousal (i.e., an increase in arousal accelerates the internal clock). The findings of this study support this hypothesis, thus proving that a high-arousal stimulus (i.e., an electrical stimulation) automatically accelerates the internal-clock system and, thereby, results in a longer perceived duration.

This study also found that the coefficient of variation of reproduced durations is larger with electrical stimulation than without electrical stimulation, which is consistent with the findings of multiple other studies [17].

## 5. Study Limitations

All participants in this study were university students. Whether the same results would be found in children and adults of other ages is worth further discussion. In this study, 0.5 s, 1.5 s, 2 s, 2.5 s, and 4.5 s were used as the target time intervals. The segmentation of short-time processing was not fully considered. It has been found that 1/3 s, 1/2 s, 1 s, and 2–3 s may be the critical points of segmentation [40,41,42]. This study only examined the impact of electrical stimulation but did not examine the impact of different intensities of electrical stimulation, which is worth further discussion in the future.

## 6. Conclusions

This study used electrical stimulation to explore the effect of negative high-arousal emotions on duration perception. This study provided evidence that the emotions induced by electrical stimulation increase the internal-clock speed, which leads to the relative overestimation of time perception.

## Figures and Tables

**Table 1:** Arousal, valence, and EDA were measured in5 target durations repeated with and without electrical stimulation.

Manage	N	Arousal		Valence		EDA	
		M	SM	M	SM	M	SM
with electrical stimulation							
0.5 s	25	5.12	0.927	4.92	0.812	1.33	0.147
1.5 s	25	5.08	0.702	4.88	0.833	1.33	0.120
2.5 s	25	5.20	0.645	4.96	0.841	1.33	0.141
3.5 s	25	5.16	0.898	4.92	0.909	1.31	0.121
4.5 s	25	5.20	0.866	4.88	0.881	1.32	0.142
without electrical stimulation							
0.5 s	25	3.96	0.735	5.52	0.872	1.03	0.113
1.5 s	25	3.84	0.746	5.60	0.913	1.05	0.130
2.5 s	25	3.76	0.723	5.56	0.712	1.02	0.143
3.5 s	25	3.80	0.816	5.56	0.583	1.02	0.107
4.5 s	25	3.80	0.645	5.52	0.586	1.02	0.118

**Table 2:** Average reproduced duration and coefficient of reproduction variation were measured in 5 target durations repeated with and without electrical stimulation.

Manage	N	Average Reproduced Duration (ms)		Coefficient of Reproduction Variation	
		M	SD	M	SD
with electrical stimulation					
0.5 s	25	491	72	0.240	0.031
1.5 s	25	1543	105	0.236	0.032
2.5 s	25	2493	129	0.242	0.025
3.5 s	25	3392	156	0.240	0.028
4.5 s	25	4768	174	0.240	0.029
without electrical stimulation					
0.5 s	25	506	66	0.274	0.039
1.5 s	25	1586	97	0.267	0.024
2.5 s	25	2520	118	0.268	0.037
3.5 s	25	3542	139	0.271	0.032
4.5 s	25	4375	151	0.269	0.034

## Data Availability

Not applicable.
